# Airway Stenting Under Venoarterial Extracorporeal Membrane Oxygenation (V-A ECMO) Support With Multimodal Sedation Including Remimazolam in a Patient With Central Airway Stenosis: A Case Report

**DOI:** 10.7759/cureus.82859

**Published:** 2025-04-23

**Authors:** Mao Kinoshita, Yu Suzuki, Masaru Shimizu, Hideya Kato, Tomomi Yamada

**Affiliations:** 1 Department of Anesthesiology, Kyoto Prefectural University of Medicine, Kyoto, JPN; 2 Department of Anesthesiology, Uji Tokushukai Medical Center, Uji, JPN

**Keywords:** airway obstruction, airway stenting, awake intubation, extracorporeal membrane oxygenation, remimazolam

## Abstract

The perioperative anesthetic management of patients with severe airway obstruction due to advanced lung cancer poses significant challenges, and no standardized protocols have been established. A 49-year-old man with severe tracheal stenosis due to extrinsic tumor compression underwent successful airway stenting supported by venoarterial extracorporeal membrane oxygenation (V-A ECMO). Due to the high risk of airway collapse, we initiated ECMO under dexmedetomidine sedation while preserving spontaneous breathing. In addition to dexmedetomidine, remimazolam then allowed safe, awake intubation and smooth induction of general anesthesia, facilitating controlled mechanical ventilation. V-A ECMO stabilizes the respiratory and circulatory functions, enabling safe, rigid bronchoscopy-guided airway stent placement. This case highlights the feasibility of combining V-A ECMO and remimazolam for high-risk airway management, demonstrating the potential benefits for patients in whom traditional induction techniques are contraindicated. Although limited to a single case, it suggests a potential role of this approach in the perioperative management of difficult airways.

## Introduction

Given the potentially poor prognosis of advanced lung cancer, the use of extracorporeal membrane oxygenation (ECMO) should be carefully evaluated on a case-by-case basis, considering both the risks and anticipated benefits [1].

Perioperative anesthetic management in patients with severe airway obstruction is challenging because it may lead to difficulties in ventilation and intubation, with a risk of acute respiratory compromise at any stage of anesthesia [2]. In particular, patients with external airway compression are at a higher risk of perioperative complications, especially when placed in the supine position during induction. Currently, no standardized protocols for perioperative management of such cases are available [3].

We report the perioperative management of a patient with central airway stenosis due to extrinsic compression from lung cancer in whom airway stenting was performed under venoarterial extracorporeal membrane oxygenation (V-A ECMO) support using remimazolam.

## Case presentation

**Figure 1:**
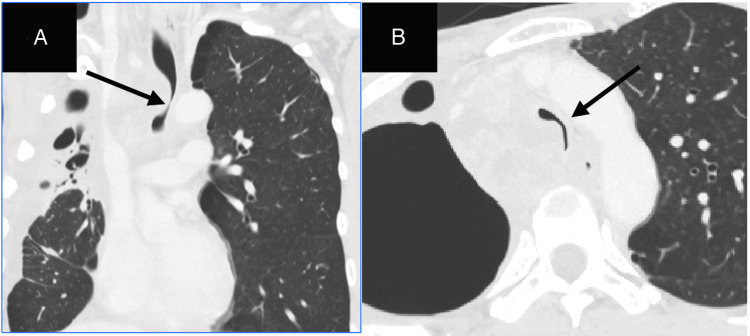
Chest computed tomography (CT) scan images Chest CT scan revealing the progression of lymph node metastases into the thoracic cavity, which caused tracheal stenosis (A). The arrow indicates the narrowest portion of the tracheal lumen, which measures approximately 4 mm in diameter (B).

Written informed consent was obtained from the patient for publication of this case report. A 49-year-old Japanese man (height, 170 cm; weight, 48 kg) underwent chemotherapy for lung cancer. The patient developed severe tracheal stenosis due to progressive paratracheal lymph node metastases from a right upper lobe lung tumor and was scheduled for airway stent placement. He presented with orthopnea and was classified as Hugh-Jones grade V. His dyspnea improved in the sitting position. His respiratory rate was 20 breaths per minute, and his peripheral oxygen saturation (SpO₂) was 95% on room air. Auscultation revealed bilaterally decreased breath sounds, a prolonged expiratory phase, and occasional wheezing. Pulmonary function testing was omitted due to marked respiratory distress. Chest computed tomography (CT) revealed that the narrowest portion of the trachea, located approximately 1 cm above the carina, had a luminal diameter of approximately 4 mm (Figure 1).

Due to the high risk of ventilation failure during induction of general anesthesia and airway stenting and the presence of tumor-related compression at the confluence of the bilateral brachiocephalic veins and superior vena cava, we planned anesthetic management with V-A ECMO support. 

**Figure 2:**
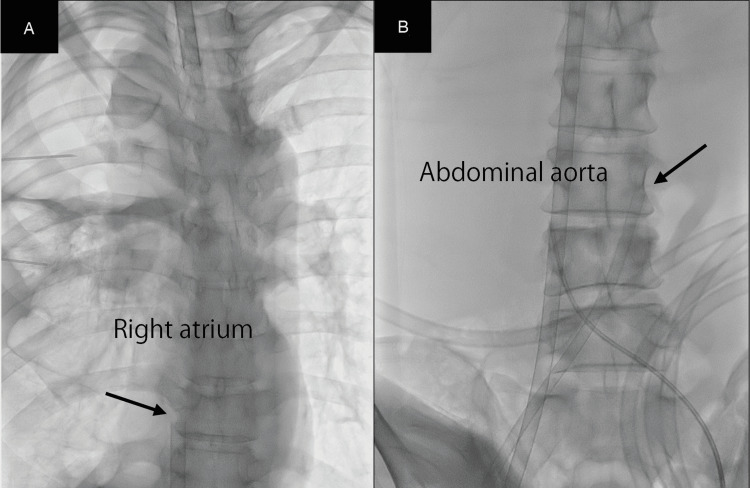
Intraoperative fluoroscopic images A 23 Fr outflow cannula was inserted into the inferior vena cava, followed by insertion of a 17 Fr inflow cannula into the right femoral artery under direct vision and fluoroscopic guidance, advancing it to the terminal abdominal aorta. The arrow indicates the outflow cannula inserted into the right femoral vein (A). The arrow indicates the inflow cannula inserted into the right femoral artery (B).

No premedications were administered. During the procedure, the patient was placed in a seated position. Monitoring included continuous electrocardiography, pulse oximetry (SpO₂), non-invasive and invasive blood pressure measurements, bispectral index (BIS; Covidien IIc, Minneapolis, MN, USA), and capnography following tracheal intubation. Invasive arterial pressure monitoring was established in both radial arteries to assess the differential pressure and monitor ECMO flow. A central venous catheter was then inserted into the left femoral vein under ultrasound guidance, with the patient placed in the supine position to the extent possible. Considering the risk of airway collapse owing to tumor compression, a venoarterial extracorporeal membrane oxygenation (V-A ECMO) circuit was prepared. Local infiltration anesthesia with 1% lidocaine with epinephrine 1:100000 was administered at the cannulation site. Dexmedetomidine was infused at a rate of 0.7 μg/kg/h, maintaining a Richmond Agitation-Sedation Scale (RASS) score between -1 and -2. The BIS remained at approximately 70. Spontaneous respiration was preserved and no body movements were observed. With the patient placed in the supine position to the extent possible, the anterior aspects of the right femoral vein and artery were surgically exposed. The right femoral vein was punctured under direct vision, and a guidewire was advanced into the right atrium. A 23 Fr outflow cannula was then inserted to the level of the inferior vena cava. Subsequently, the right femoral artery was punctured under direct vision, and under fluoroscopic guidance, a 17 Fr inflow cannula was inserted and advanced to the terminal portion of the abdominal aorta (Figure 2).

**Figure 3:**
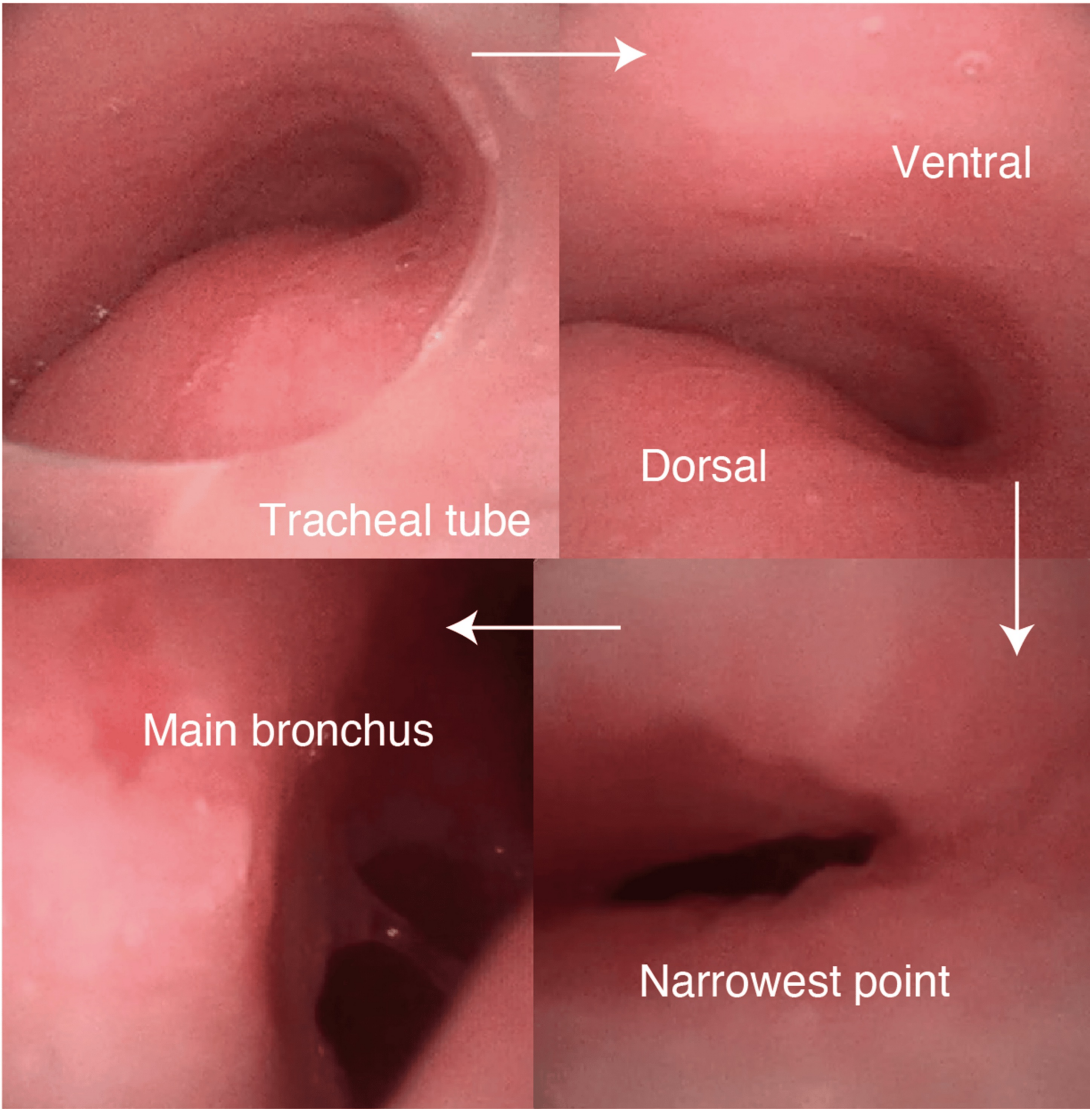
Bronchoscopic findings The images sequentially demonstrate the progression of the bronchoscope into the distal airway.

V-A ECMO was initiated, with the total flow reaching a maximum of 3.5 L/min and subsequently maintained at approximately 2.0 L/min. Arterial blood gas analysis of the right radial artery revealed a partial oxygen (PaO₂) of 56 mmHg. Intraoperatively, the position of the cannula was confirmed under fluoroscopic guidance, the patient was asked to open her mouth, and 4 mL of 4% lidocaine was sprayed from the tongue base to the pharynx. Under bronchoscopic guidance, 2 mL of 4% lidocaine (lidocaine hydrochloride; AstraZeneca, Tokyo, Japan) was administered to the laryngopharynx and 2 mL to the trachea. A bronchoscope (Ambu® aScope 4™ Broncho, Ambu A/S, Baltorpbakken, Denmark) was inserted beyond the vocal cords to identify the tracheal stenosis, and an 8.0 mm internal diameter endotracheal tube was placed proximal to the stenotic segment while preserving spontaneous respiration (Figure 3).

**Figure 4:**
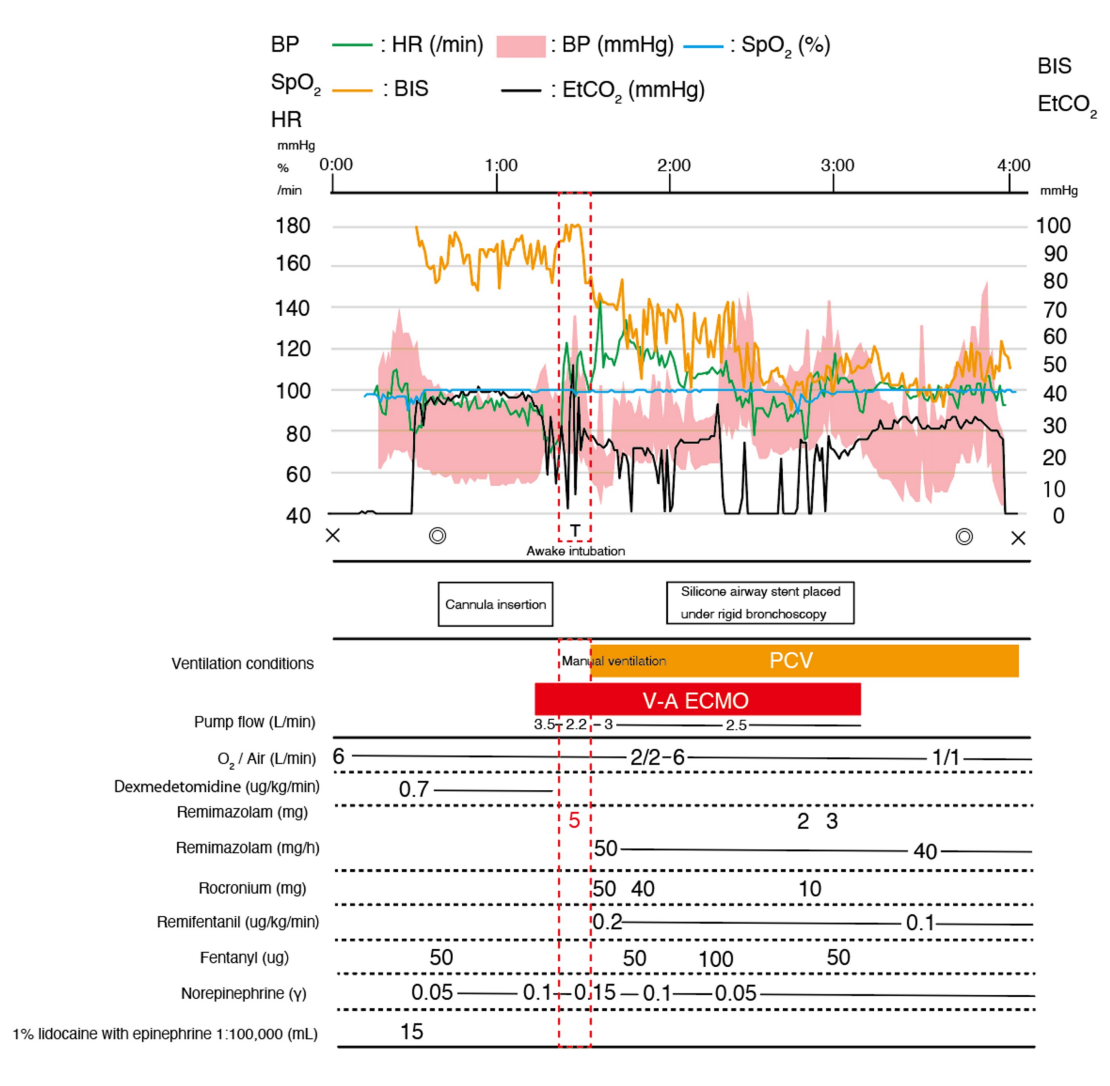
Intraoperative anesthesia record of the case ×, start and end of anesthesia; ◎, start and end of the operation; T, tracheal intubation; HR, heart rate; ­ SpO_2_, percutaneous oxygen saturation; BP, blood pressure; EtCO_2_, end-tidal carbon dioxide partial pressure; BIS, bispectral index; PCV, pressure-controlled ventilation; V-A ECMO, venoarterial extracorporeal membrane oxygenation.

Following endotracheal intubation, a continuous infusion of remimazolam and remifentanil was initiated. According to the prescribing information for remimazolam, a continuous intravenous infusion at 12 mg/kg/h is recommended until loss of consciousness is achieved. In this case, general anesthesia was induced with an initial intravenous bolus of remimazolam (0.2 mg/kg, 5 mg) and a continuous infusion of remifentanil at 0.2 μg/kg/min. Loss of consciousness occurred approximately 30 seconds after remimazolam administration, while spontaneous respiration was maintained. After confirming that ECMO total flow was stable, a neuromuscular blocking agent was administered in consultation with the surgical and clinical engineering teams. Endotracheal intubation was then performed, and anesthesia was maintained with continuous infusions of remimazolam at 1 mg/kg/h and remifentanil at 0.2 μg/kg/min. Mechanical ventilation was initiated in pressure-controlled ventilation (PCV) mode with an FiO₂ of 1.0. Adequate ventilation was achieved with a peak airway pressure of 25 cmH₂O and a tidal volume of 250-300 mL (Figure 4). Manual ventilation was provided during the transition until spontaneous breathing ceased. Following rocuronium administration, no worsening of the anticipated airway stenosis was observed. Tidal volume and SpO₂ remained stable, indicating preserved ventilation. Pressure-controlled mechanical ventilation was then continued without signs of inadequate gas exchange. The subsequent increases in opioid and neuromuscular blocker dosages reflected intraoperative responses to surgical stimuli, not insufficient hypnotic depth from remimazolam.

**Figure 5:**
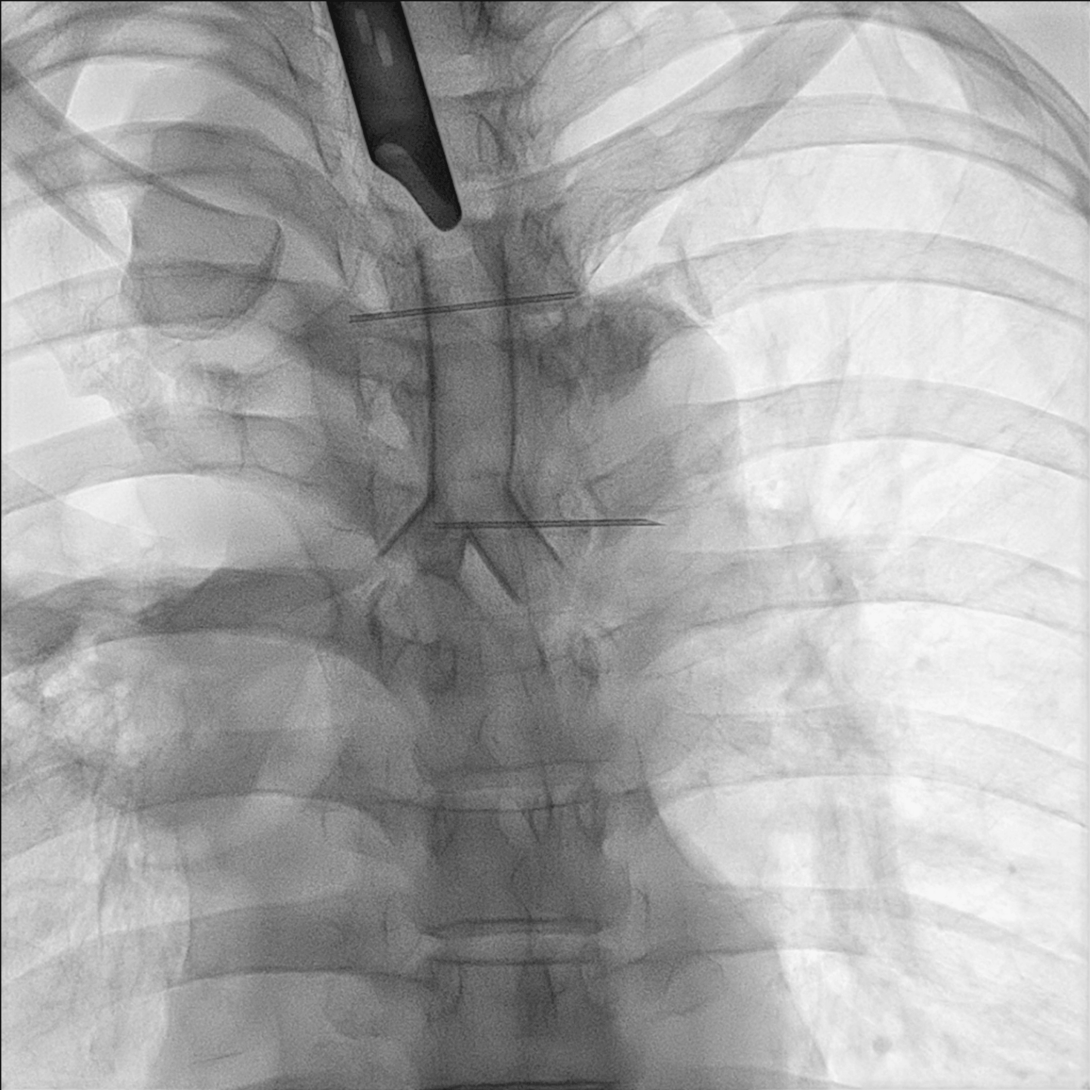
Chest X-ray imaging Chest X-ray showing a silicone stent inserted at the carina using a rigid bronchoscope.

Circulatory and respiratory management was achieved by adjusting the ECMO flow and applying controlled mechanical ventilation. Following balloon dilation of the stenotic segment, a silicone airway stent (Dumon®, Novatech SA, La Ciotat, France) was placed under rigid bronchoscopy (Figure 5).

During the procedure, SpO₂ remained stable at 100% and the CO₂ level was approximately 30 mmHg. FiO₂ was maintained at 0.6 throughout anesthesia and after insertion of the bronchoscope. The operative duration was 181 min, blood loss was 3 g, and the total duration of V-A ECMO support was 116 min. After the procedure, the patient was weaned off ECMO and transferred to the intensive care unit (ICU) while still being intubated. The patient was extubated on postoperative day 1 and was discharged in a stable respiratory condition on day 6. 

## Discussion

This case report highlights two important findings: the feasibility of performing sedated intubation with preserved spontaneous breathing using remimazolam after V-A ECMO initiation and the utility of V-A ECMO support in stabilizing respiratory and circulatory function during airway stenting in a patient with severe central airway obstruction.

First, remimazolam enables safe and effective intubation during spontaneous breathing following ECMO cannulation under dexmedetomidine sedation. Regarding the ECMO cannulation and awake intubation phases, remimazolam was intentionally withheld to preserve airway reflexes and patient cooperation. Instead, dexmedetomidine was selected for its ability to provide light sedation while maintaining spontaneous breathing and neurological responsiveness-factors critical for procedural safety and patient communication. For ECMO cannulation, we opted for minimal sedation to maintain hemodynamic and neurological stability. In high-risk airway cases, where general anesthesia induction poses a risk of complete airway obstruction, preserving spontaneous ventilation is crucial [4]. Remimazolam, with its favorable pharmacokinetic profile and the availability of a reversal agent, flumazenil, provides a controllable sedation strategy with minimal respiratory depression. Following intubation and initiation of mechanical ventilation, the patient remained on V-A ECMO. In this context, we considered that intravenous anesthesia would be more appropriate than inhalational agents, as gas exchange is markedly altered and the uptake of volatile agents can be unpredictable due to circuit-related absorption. Although alternative intravenous agents such as propofol were considered, we opted to continue remimazolam to ensure seamless anesthetic management. Since remimazolam had already been used for induction with favorable effects-including stable hemodynamics and preservation of spontaneous breathing, its continued use allowed us to maintain a consistent depth of anesthesia without the need to switch agents intraoperatively. Recent studies have reported the safe and effective use of remimazolam combined with flumazenil in rigid bronchoscopy procedures, demonstrating favorable recovery and sedation profiles [5]. This approach allowed for stable oxygenation, smooth intubation, and a seamless transition to mechanical ventilation [6].

Second, V-A ECMO provides effective perioperative support for respiratory and circulatory management during airway stent placement. Given severe tracheal narrowing and positional orthopnea, ECMO support ensured adequate gas exchange and hemodynamic stability during anesthesia and surgical manipulation. This approach minimizes the risk of ventilatory failure and allows controlled stent deployment under rigid bronchoscopy [7,8]. Literature supports the effectiveness of ECMO in providing respiratory and circulatory support during airway stent placement. Several case series and retrospective studies have demonstrated the safety and efficacy of ECMO-assisted airway stenting, particularly in cases of malignant central airway obstruction and severe tracheal stenosis requiring rigid bronchoscopy [9-12]. However, the choice between venovenous (V-V) and venoarterial (V-A) ECMO should be tailored to the patient's condition and specific requirements of the procedure. In addition to the primary management goals, the intraoperative care in this case included attention to the distribution of oxygenated blood between the native cardiac output and ECMO flow. To prevent cerebral hypoxia, the differential arterial pressure and pulmonary oxygenation status were carefully monitored throughout the procedure.

Taken together, these findings suggest that combining V-A ECMO support with remimazolam-based anesthesia may be a viable strategy in selected patients with critical airway obstruction in whom conventional induction of general anesthesia is unsafe. This approach may be particularly useful in thoracic oncology or interventional pulmonology settings involving extrinsic airway compression. Although ECMO has been used in high-risk airway procedures, the present case is the first reported instance of tracheal stenting performed under rigid bronchoscopy with remimazolam anesthesia and V-A ECMO support.

This study had several limitations. First, as this was a single-case report, the findings are not broadly generalizable. Second, successful management requires significant institutional expertise in ECMO and close coordination among the anesthesiology, surgery, and perfusion teams. Third, although the mixing-point dynamics were monitored, real-time objective assessment was limited, which may have affected the reproducibility of such nuanced intraoperative management.

## Conclusions

In this case, multimodal sedation including remimazolam and remifentanil allowed for safe ECMO cannulation and airway stenting in a patient with severe tracheal stenosis. While awake ECMO initiation using low-dose opioids is possible, we selected this strategy to ensure adequate sedation, hemodynamic stability, and patient cooperation throughout the procedure.
